# Color Phenomena Associated With Energy Transfer in Afterglows and Atomic Flames

**DOI:** 10.6028/jres.067A.041

**Published:** 1963-08-01

**Authors:** Arnold M. Bass, H. P. Broida

## Abstract

Reactions involving reactive species produced in electric discharges are frequently characterized by the emission of visible light of many different colors. Some typical afterglows and atomic flames have been photographed, and the observed colors (or spectral distributions) are discussed with regard to the reactions from which they arise. Laboratory studies of this sort are helpful for the understanding of the energy transfer processes which occur in flames, in electrical discharges, and in the upper atmosphere.

## 1. Introduction

Brightly colored afterglows from electric discharges have provided a source of laboratory demonstrations and wonder for nearly 80 years. In 1884 Warburg [1]^1^ noticed that, in a tube of nitrogen at low pressure which was subjected to an electric discharge, glows persisted for a long time after the exciting voltage was removed. Since the beginning of this century, afterglows in nitrogen [2] and oxygen [3] have been investigated extensively. Most of the phenomena associated with these afterglows are caused by reactions of atomic species [4]. It also has been found that under some conditions, in products of electric discharges condensed as solids at very low temperatures, there is an “afterglow” from the solid [5, 6].

When dissociation products from an electric discharge through gases at low pressure (0.01 to 100 mm Hg) are mixed with suitably reacting gases or vapors, bright regions known as atomic flames are observed [7]. In addition to the visible light which is observed and discussed below, there is also ultraviolet and infrared emission. Spectra of flames obtained on mixing hydrocarbons with atomic oxygen [8, 9] have been used as a method to help in the understanding of reaction zones in combustion processes. Emission spectra of brilliant glows obtained at the point of mixing of certain organic compounds with atomic nitrogen have been studied [10, 11] and used to obtain information about the reactions [12, 13] taking place. There is little emission from reactions taking place with hydrogen atoms [7, 8] but weak flames are observed from mixing ozone with hydrogen atoms, and much brighter flames from ozone mixed with both hydrogen and nitrogen atoms [14].

Very few studies have been made of glows which occur in short times after the discharge. However some observations have been made as part of wind tunnel visualization efforts [15].

**Figure 1:**
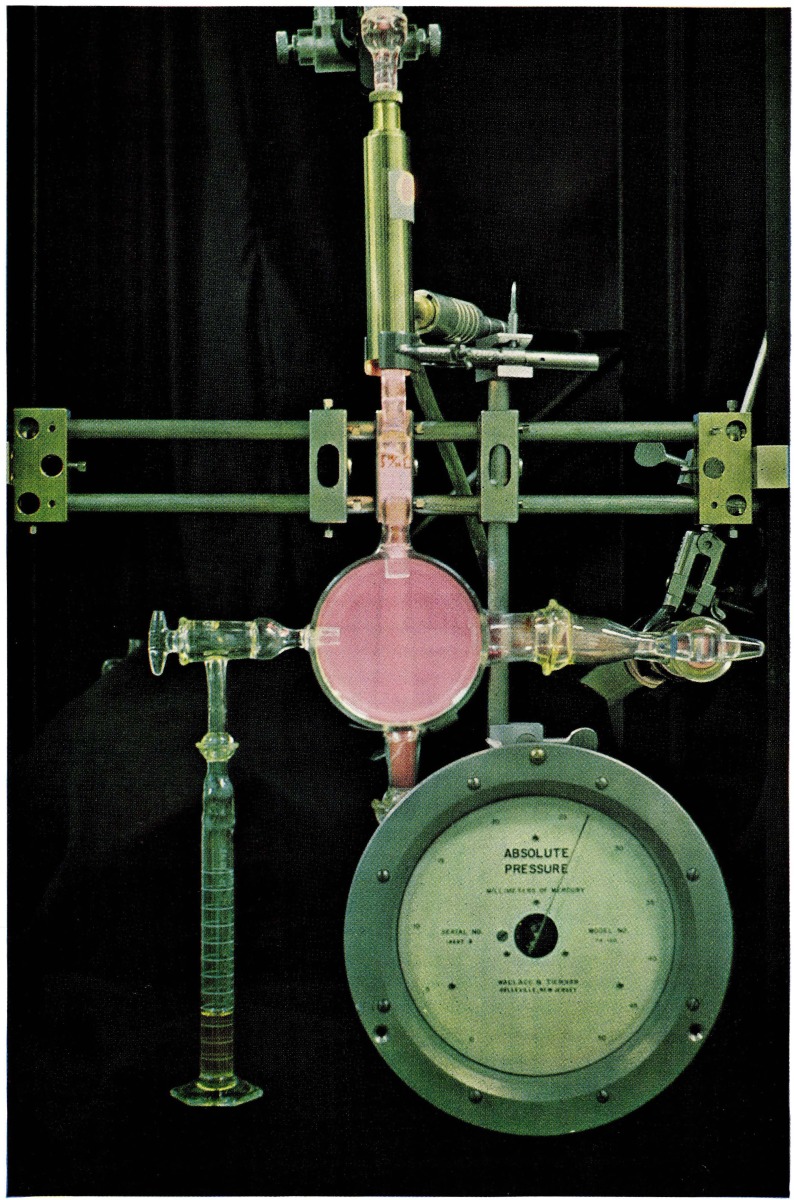
Apparatus for producing afterglows and atomic flames. The pink glow in the cylindrical vessel is a nitrogen afterglow in an excess of helium at a pressure of 27 mm Hg.

**Figure 2:**
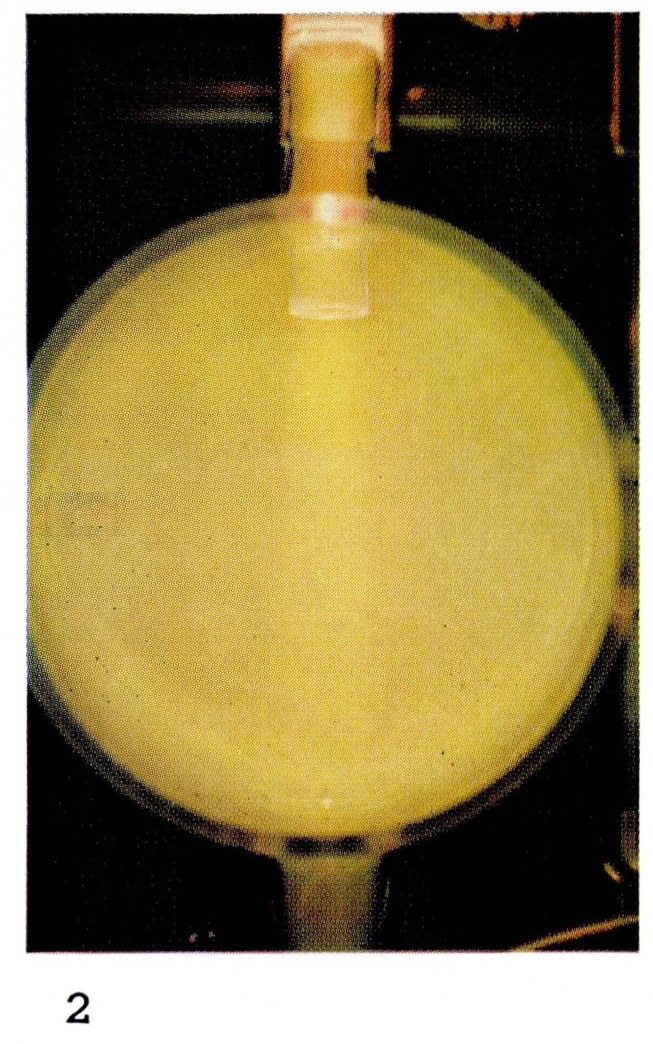
Afterglows from Discharges Through Nitrogen Pure nitrogen at 4 mm Hg, pressure showing the typical Lewis-Rayleigh afterglow.

Previous attempts to obtain color photographs of these phenomena have met with only partial success. However, recent developments in photographic techniques and color film, as well as improved control of the afterglow and flame systems have made possible the satisfactory photographic recording of these glows. A group of photographs of characteristic glows and flames has been assembled in figures 2 through 9, and 11 through 24; figures 1 and 10 show the apparatus used for the excitation of the glows.

## 2. Experimental Procedure

Afterglows and atomic flames were photographed in the apparatus shown in figure 1. High purity gases from cylinders were admitted to the discharge region by means of control valves visible near the top of the picture. An electrodeless discharge was maintained in a quartz tube of 10 mm i.d. by means of a microwave cavity operated at 2450 Mc/s with a maximum input power of 125 w. With this arrangement, glows could be observed at pressures between 0.1 and 100 mm Hg. The brightest glows occurred at pressures between 1 and 10 mm Hg. Two different gases could be separately admitted and mixed before the discharge. A side tube in the cylindrical chamber (9.5 cm diameter) allowed the mixing of another gas downstream from the discharge. Another inlet tube allowed pressure measurements to be made with an aneroid type, direct reading manometer. A pumping line (2.5 cm diameter) was connected to a high speed, mechanical vacuum pump.

In order to observe very short time phenomena the apparatus shown in figure 10 was used. In this apparatus, which was made of Pyrex glass, the electric discharge (2450 Mc/s) occurred in a converging-diverging (de Laval) nozzle with a throat of about 2 mm diam and an exit of 6 mm. The gases were emptied into a 9 cm diam chamber, 100 cm long. A vacuum pump with a capacity of 500 liters/sec was used. Cleanliness of the apparatus and of the gases were essential for many of the phenomena which are pictured in this paper.

The critical factor for the reproducible photography of these phenomena is the correct control of exposure time. This was achieved through the use of a Honeywell Pen tax 3°/21° exposure meter which has very high sensitivity for an acceptance angle of 3°. The narrow acceptance angle permits exposure readings from restricted regions of the flames and thus adjustments may readily be made for small changes in brightness. These photographs were made on Kodachrome II film (daylight type) rated at an exposure index of 32. Exposure times were varied from 1/25 sec to 30 sec. Changes of exposure time by as little as a factor of 2 made the difference between good and poor color reproduction. For the longer exposures attempts were made to take account of the reciprocity-law failure by the use of color correction filters as recommended by the film manufacturer. However, it was found that the best color reproduction was obtained without the use of filters.

## 3. Discussion

Two general methods have been used in the study of afterglows. In a static gas system after pulsing an electrical discharge, changes of color can be observed as function of time. Alternatively, the same phenomena can be observed in a flowing gas system as a function of distance downstream from the discharge. The observed phenomena are caused by various reactions of energetic species. Different color regions indicate different concentrations of atoms, molecules, and ions and the collision processes occurring between these species.

There are many known species in discharges in pure atomic and diatomic gases: atoms and diatomic molecules each in several electronic states, ions of atoms and molecules, electrons, and photons. Conditions in the discharge affect the relative concentrations of these species. Concentrations change as a function of time after the discharge with most of the more energetic species decreasing monotonically with time. However in some cases, the collision processes cause increases of certain species. In nitrogen at times a few milliseconds after the discharge, the number of ions [16] and of electronically excited molecules is increased [17], (see fig. 6). A very energetic N_4_ molecule has been postulated as the energy carrier in this case [18]. At times of tens of microseconds after a discharge in very pure helium, there is a large increase in the concentration of electronically excited helium atoms and diatomic helium molecules [19], (see fig. 13). The details of the energy exchange mechanisms involved in these short-time afterglows are not yet understood.

Some collision processes occurring in afterglows have been carefully studied. For example, the long-time nitrogen afterglow is understood in terms of reactions of nitrogen atoms to form molecular nitrogen [20]:

$$N\left({\;}^{4}S\right)+N\left({\;}^{4}S\right)+M\to N_{2}\left({\;}^{5}\Sigma_{g}^{+}\right)+M$$ (1)

M is any third body in the three body collision process. The electronically excited molecule of nitrogen, $N_{2}\left({\;}^{5}\Sigma_{g}^{+}\right)$ can undergo a collision to change its electronic state to the upper state of the first positive system of nitrogen, N_2_(B ^3^Π*_g_*):

$$N_{2}\left({\;}^{5}\Sigma_{g}^{+}\right)+M\to N_{2}\left(B^{3}\Pi_{g}\right)+M.$$ (2)

This is followed by radiation of a photon, *hv*:

$$N_{2}\left(B^{3}\Pi_{g}\right)\to N_{2}\left(A^{3}\Sigma_{u}^{+}\right)+hv.$$ (3)

Because the energy available in reaction (1) is equal to the dissociation energy of nitrogen, 9.76 ev (plus a small addition of kinetic energy of the colliding particles), the total energy of the B ^3^Π*_g_* molecular nitrogen is limited to this amount. As a consequence, the twelfth vibrational level of the B ^3^Π*_g_* state is the highest one that can be populated. The emission from reaction (3) is the First Positive system of N_2_ with a non-Boltzmann distribution of vibrational populations, characterized in the visible region by a maximum near *v*′= 10.

**Figure 3:**
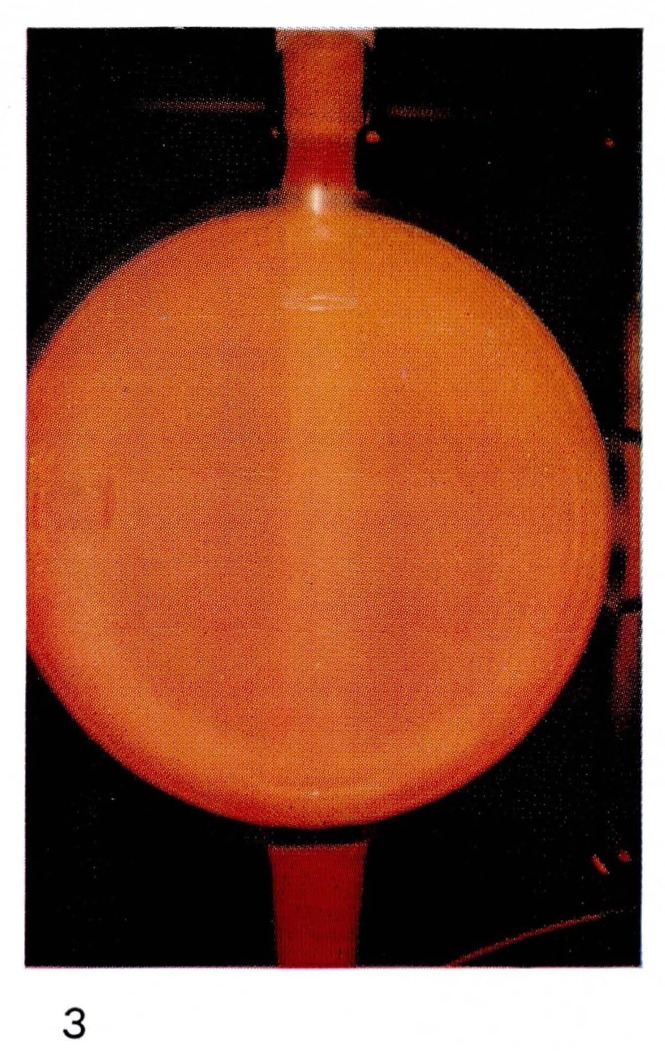
Afterglows from Discharges Through Nitrogen Same amount of nitrogen in large excess of helium at 50 mm Hg.

It is this particular emission of the First Positive system of N_2_ which gives the “straw-yellow” color of figures 2 and 11 to the Lewis-Rayleigh nitrogen afterglow. If the nitrogen is diluted with large amounts of a rare gas such as helium, the population distribution in the N_2_(B ^3^Π*_g_*) state is changed [21] moving to maximum populations near *v*′=8. This corresponds to a shift toward the red end of the spectrum and is observed visually as a change in color of the afterglow. The change in color can be seen by comparing figures 2 and 3, and figures 11 and 12.

**Figure 4:**
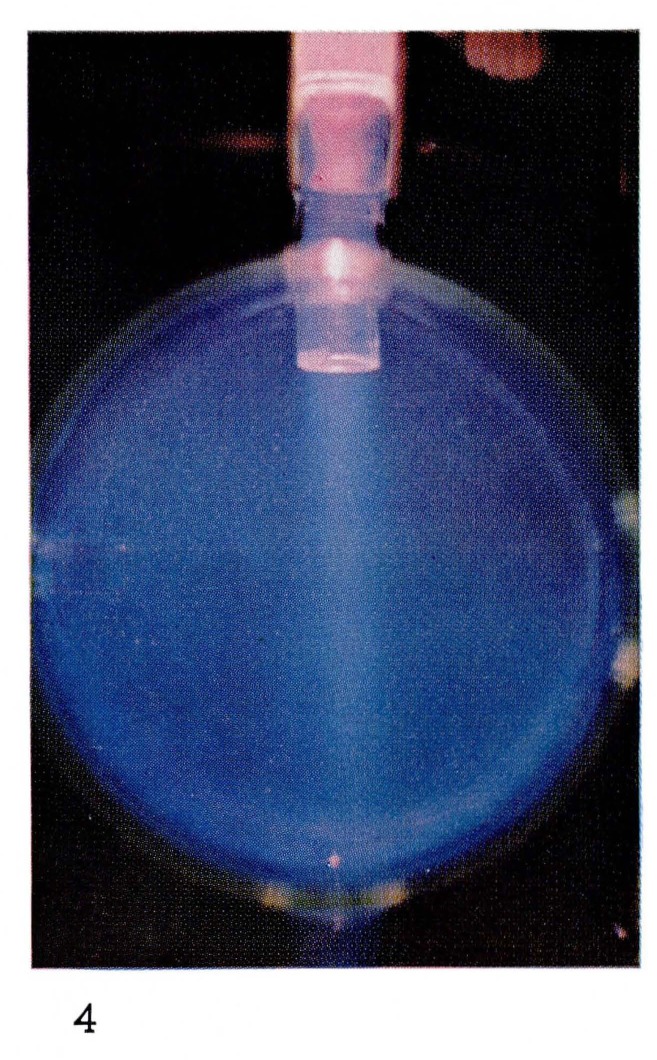
Afterglows from Discharges Through Nitrogen Approximately 1 percent O_2_ added to the nitrogen of figure 2.

Impurities in the discharge cause large changes in concentrations and in the kinds of species. Additions of small amounts of oxygen cause large changes in the colors of the afterglow because of reactions with oxygen atoms formed in the discharge. Oxygen atoms can react with nitrogen producing NO molecules in the upper state of the *β* bands of NO, B ^2^Π:

$$N\left({\;}^{4}S\right)+O\left({\;}^{3}P\right)+M\to \text{NO}\left({\text{B}\;}^{2}\Pi \right)+M$$ (4)

followed by

$$\text{NO}\left({\text{B}\;}^{2}\Pi \right)\to \text{NO}\left({\text{X}\;}^{2}\Pi \right)+hv$$ (5)

where *hv* represents radiation which is brightest in the blue region of the spectrum [3, 22], (fig 4). Nitrogen atoms react rapidly with the NO to make N_2_ and oxygen atoms:

$$N\left({\;}^{4}S\right)+\text{NO}\to N_{2}+O\left({\;}^{3}P\right)$$ (6)

When sufficient oxygen is added all the nitrogen atoms are consumed in the reactions. In the absence of nitrogen atoms, NO reacts with oxygen atoms to give NO_2_ in an electronically excited state [23] and this is followed by the emission of a photon.

$$\text{NO}+O\left({\;}^{3}P\right)+M\to {\text{NO}}_{2}^{*}+M$$ (7)

$${\text{NO}}_{2}^{*}\to {\text{NO}}_{2}+hv.$$ (8)

**Figure 5:**
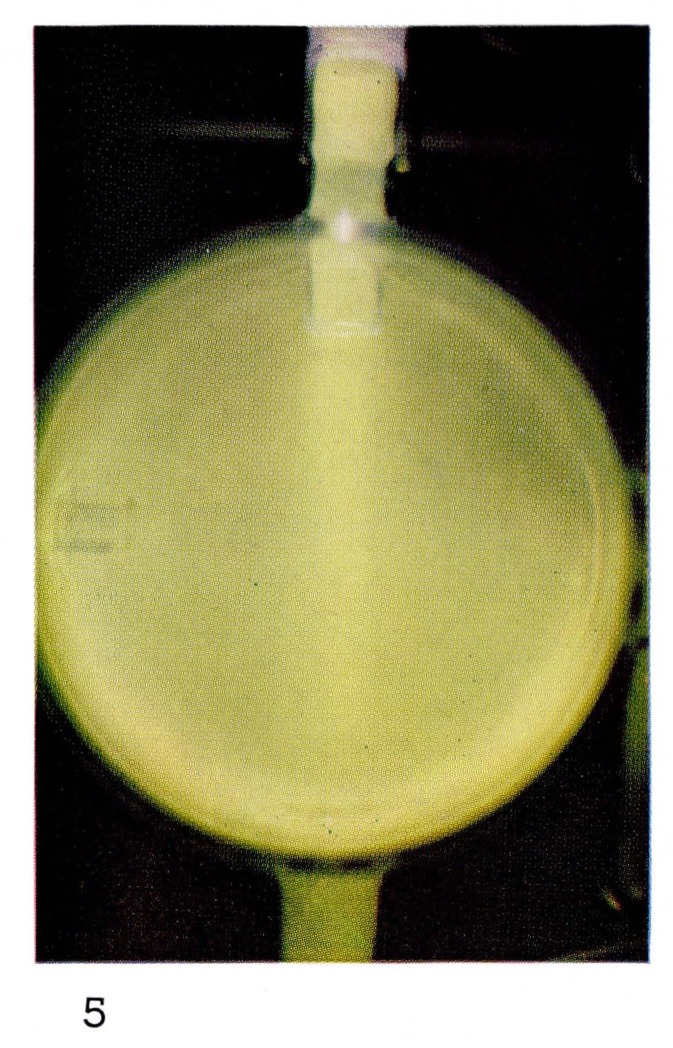
Afterglows from Discharges Through Nitrogen Approximately 3 percent O_2_ added to the nitrogen, showing the “air-afterglow.”

**Figure 6:**
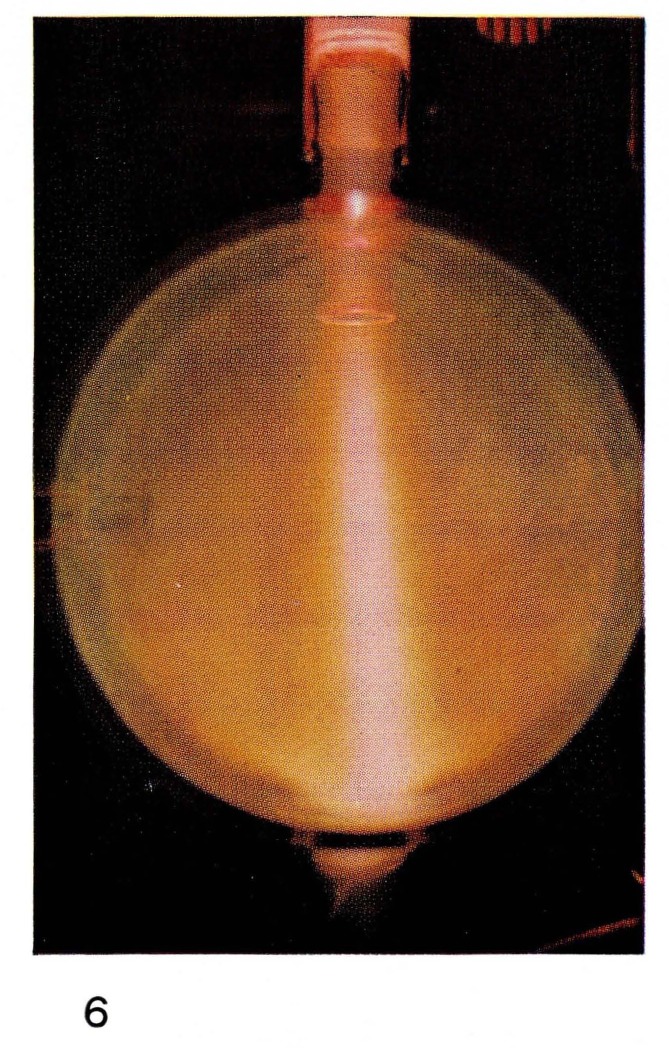
Afterglows from Discharges Through Nitrogen Short-duration, pink “auroral” afterglow surrounded by the Lewis-Rayleigh afterglow at 4 mm Hg.

Reaction (8) causes the yellow green “air afterglow” shown in figure 5.

**Figure 7:**
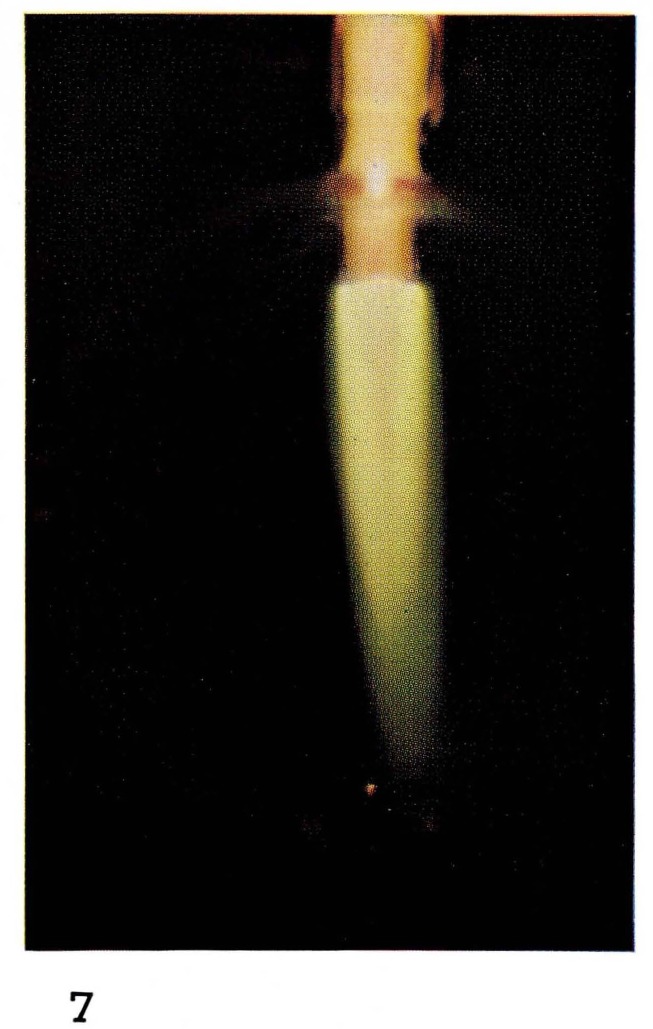
Atomic Flames Nitrogen atoms with added nitric oxide (NO).

**Figure 8:**
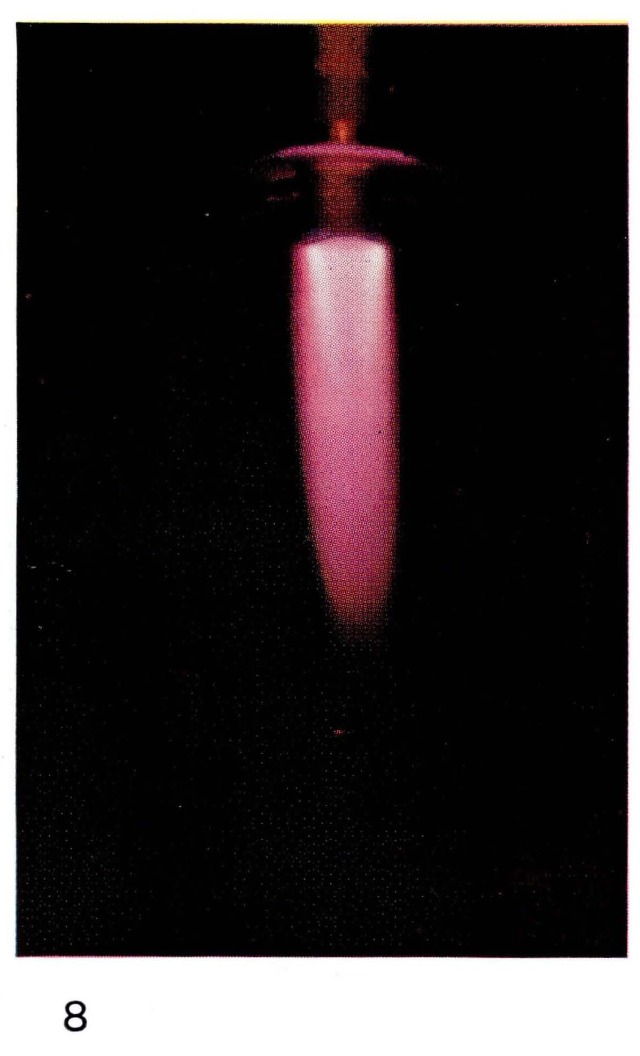
Atomic Flames Nitrogen atoms with added methylene chloride (CH_2_Cl_2_).

**Figure 9:**
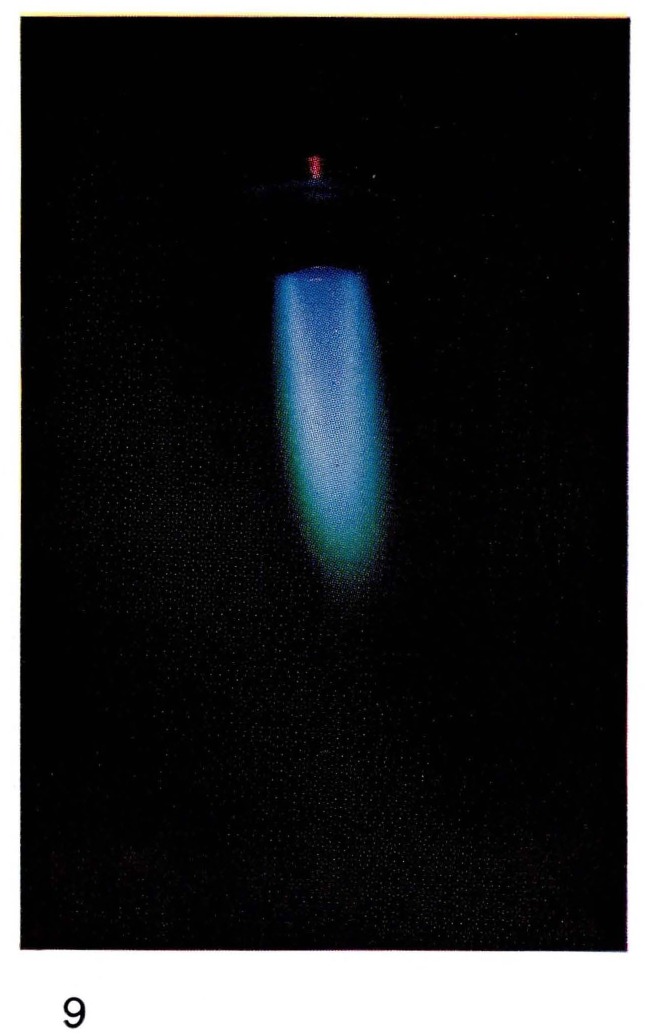
Atomic Flames Oxygen atoms with added acetylene (C_2_H_2_).

**Figure 10:**
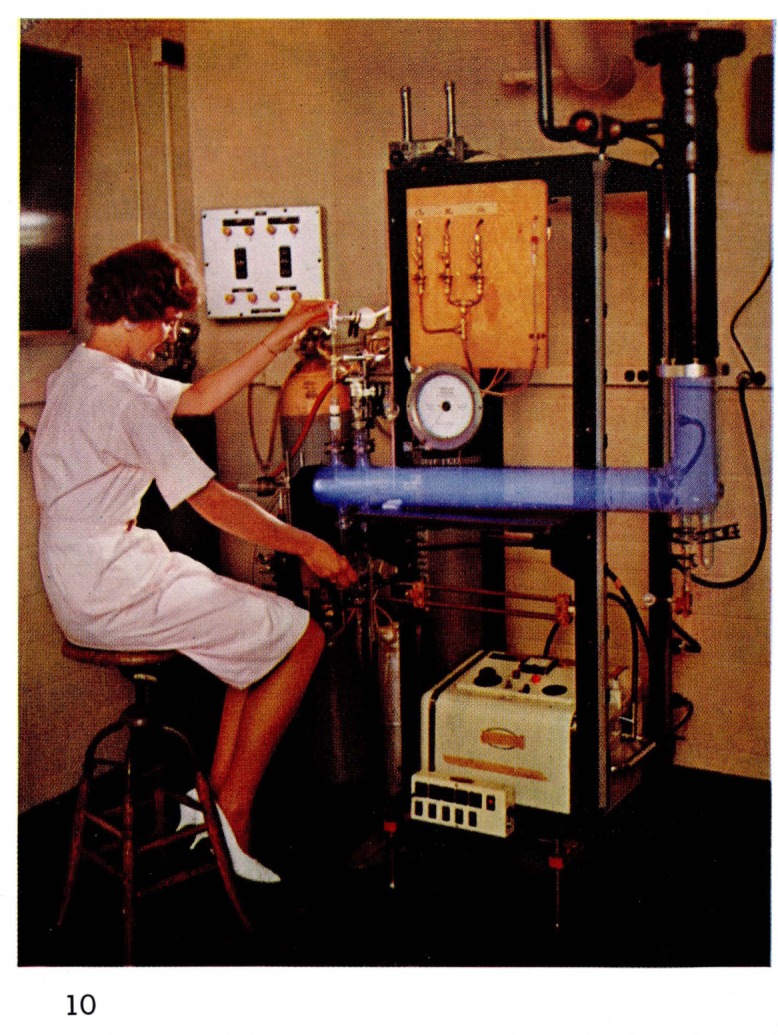
Atomic Flames Astrojet apparatus for producing helium afterglows and flames.

**Figure 11:**
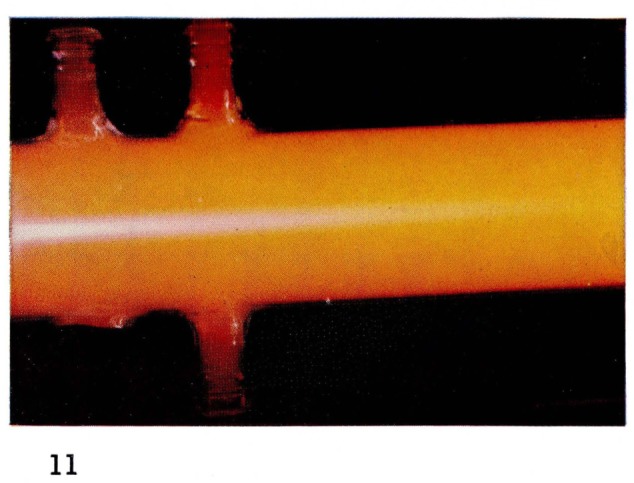
Afterglows from Discharges Through Nitrogen in the Astrojet Pure nitrogen.

**Figure 12:**
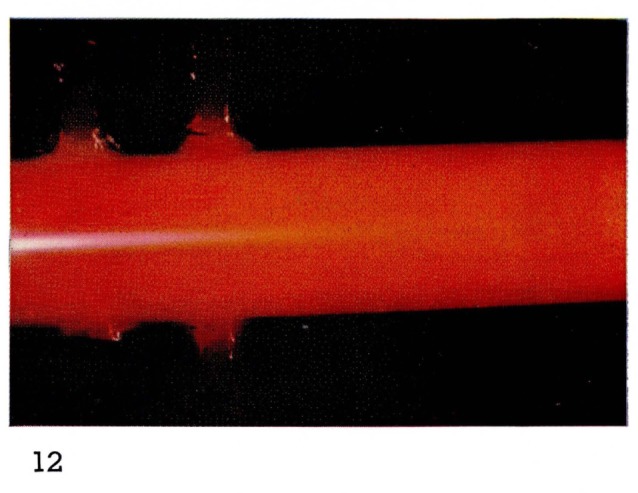
Afterglows from Discharges Through Nitrogen in the Astrojet Nitrogen with large excess of helium.

**Figure 13:**
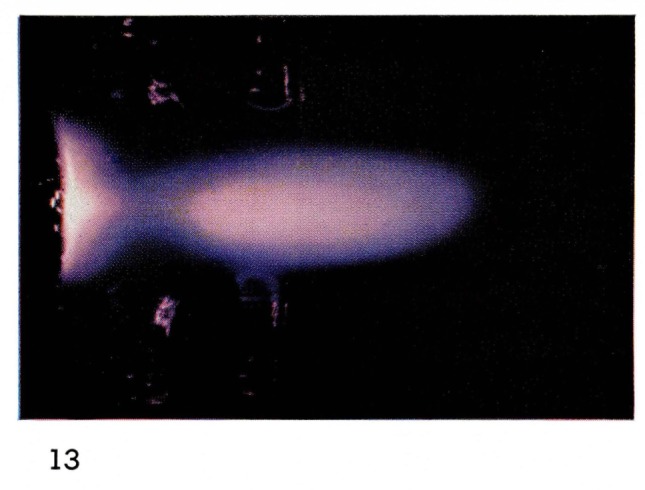
Short·Duration Glows from Discharges Through Helium in a deLaval Nozzle Pure helium afterglow at 1.4 mm Hg.

Bright “atomic flames” often can be seen when gases or vapors are added to the atoms produced in the discharge. For example the glow produced by reaction (8) is formed by adding NO in excess of the amount of nitrogen atoms to the afterglow of pure nitrogen. Thus the color, and the spectral emission, of figures 5 and 7 are the same. If hydrocarbon vapors are added to nitrogen, as in figures 8 and 22, bright visible emission caused predominatly by CN is observed [10, 11, 13]. Atomic oxygen mixed with hydrocarbons shows a completely different appearance (figs. 9 and 21) because the emission is due to CH and C_2_ [8, 9].

**Figure 14:**
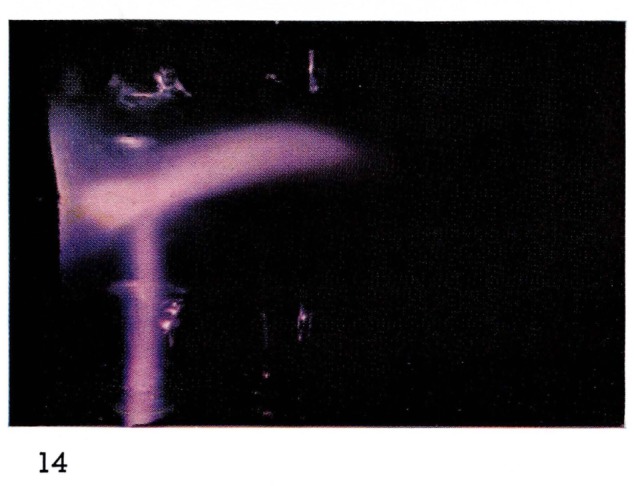
Short·Duration Glows from Discharges Through Helium in a deLaval Nozzle Intersection of two high-velocity gas streams each showing the pure helium afterglow.

**Figure 15:**
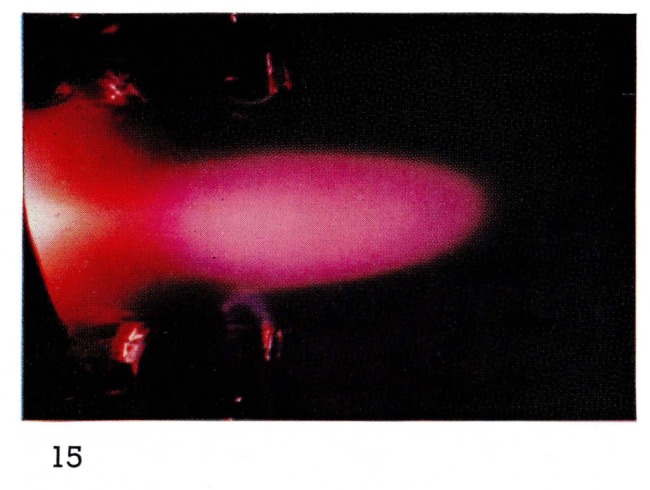
Short·Duration Glows from Discharges Through Helium in a deLaval Nozzle Same conditions as 13 (1.4 mm Hg) with the addition of less than 0.01 percent neon downstream from the discharge.

**Figure 16:**
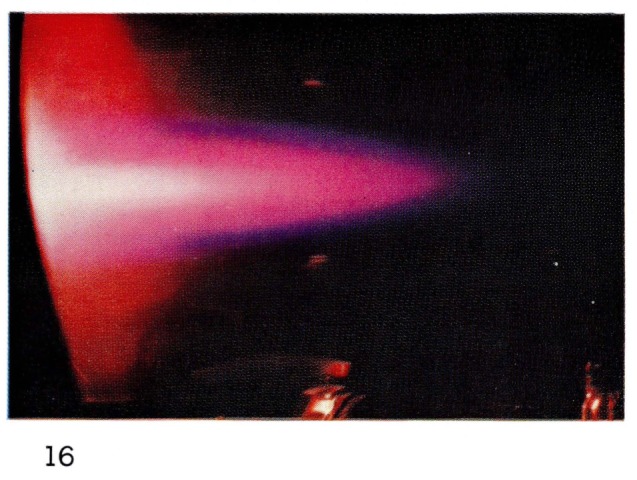
Short·Duration Glows from Discharges Through Helium in a deLaval Nozzle Same mixture as 15 at 10 mm Hg.

To observe steady-state afterglows at very short times after a discharge, it has been necessary to use supersonic flows from a discharge in a de Laval nozzle [15, 19]. At such high gas velocities, or rather in such short times, there is very little mixing of gases as illustrated in figure 14. However it is possible to observe some very efficient collision processes with metastable helium triplet state atoms. Energy exchange collisions with neon cause excitation of many electronic states of neon (figs. 15 and 16). Oxygen and nitrogen are ionized as well as electronically excited by collisions with metastable helium atoms, by what is known as the Penning process [24]:

$$\text{He}\;\left(2^{3}S\right)+N_{2}\to N_{2}^{+}+\text{He}\left(1{\;}^{1}S\right)+e$$ (9)

$$\text{He}\left(2^{3}S\right)+O_{2}\to O_{2}^{+}+\text{He}\left(1{\;}^{1}S\right)+e$$ (10)

**Figure 17:**
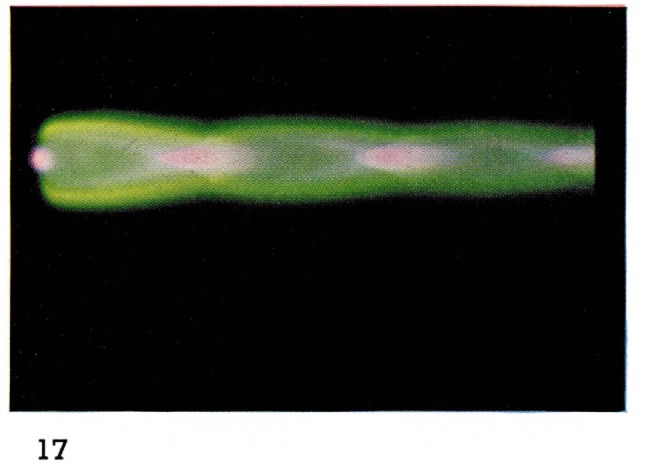
Helium Flames at Supersonic Velocities Metastable helium at 2 mm Hg with oxygen added downstream from the discharge.

**Figure 18:**
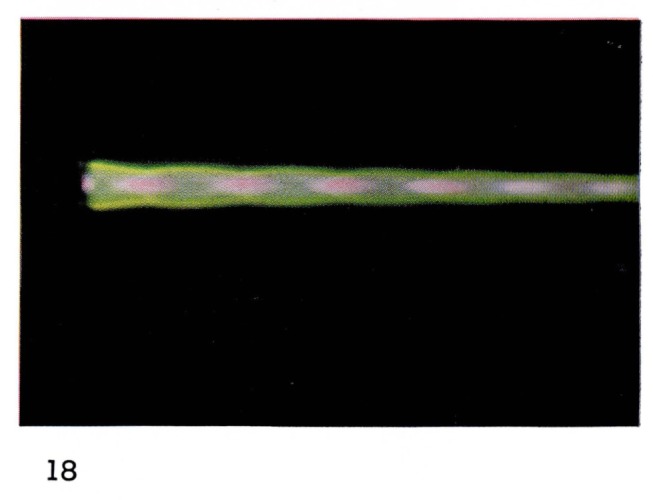
Helium Flames at Supersonic Velocities Same mixture as 17, at 4 mm Hg.

**Figure 19:**
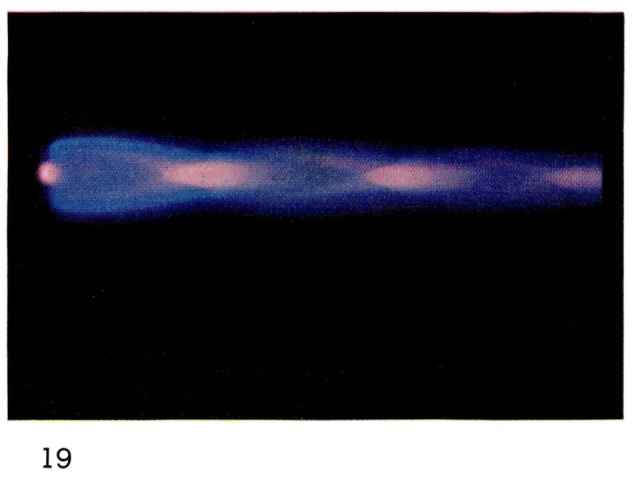
Helium Flames at Supersonic Velocities Metastable helium at 2 mm Hg with oxygen added downstream from the discharge.

**Figure 21:**
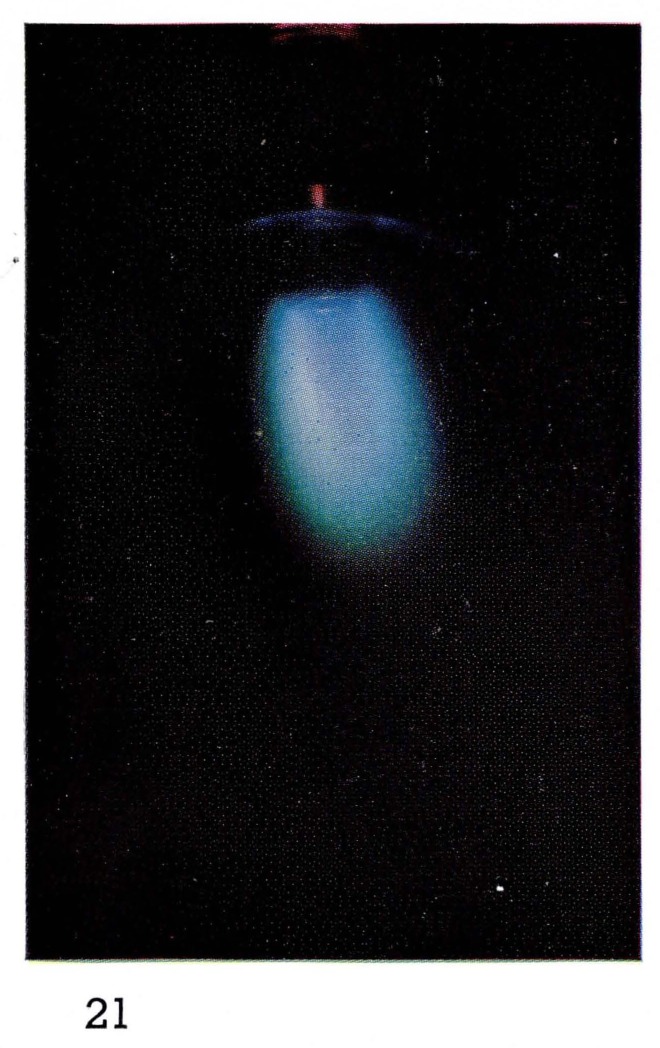
Flames Atomic oxygen with added acetylene (C_2_H_2_).

**Figure 22:**
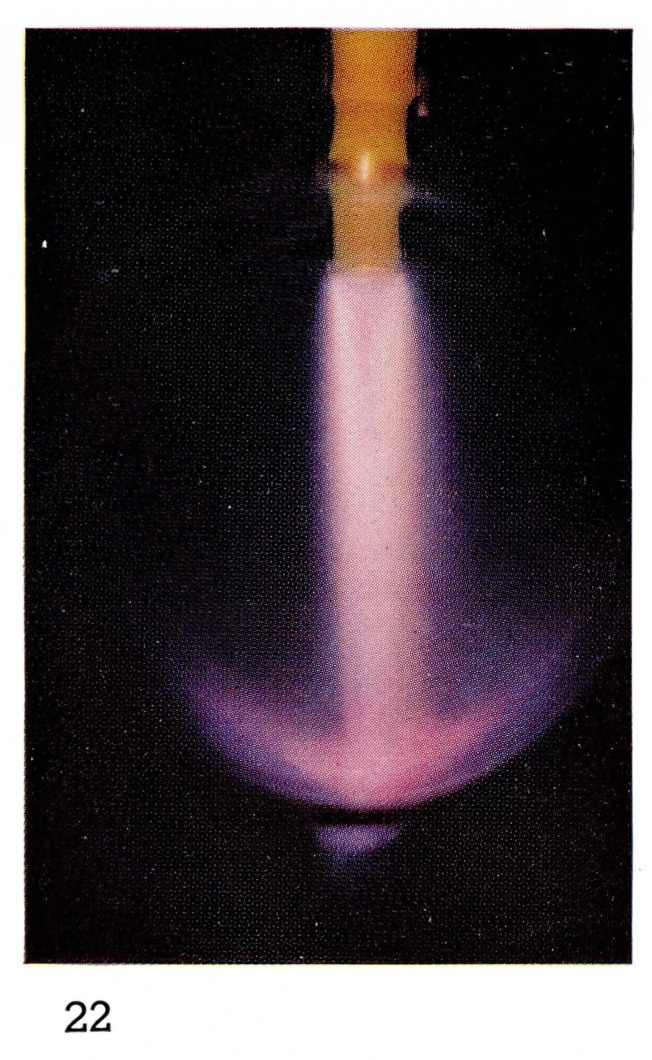
Flames Atomic nitrogen with added methylene chlorine (CH_2_Cl_2_).

Some dissociation of molecules also occurs since emission of atomic oxygen has been observed [19]. In figures 17, 18, and 19 the zones in the flames in which the various species are excited are clearly distinguishable from the colors: atomic and molecular helium is pink; $N_{2}^{+}$ is blue; $O_{2}^{+}$ and O are green. The diamond-shaped regions in which the different species appear are characteristic of shock fronts in the supersonic flow regime.

## Figures and Tables

**Figure 20:**
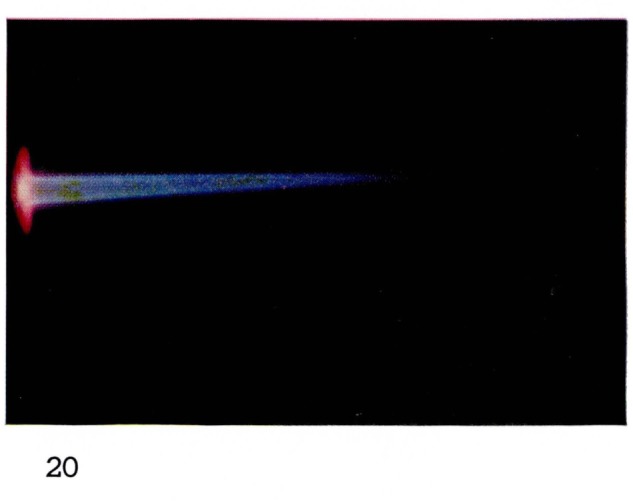
Helium Flames at Supersonic Velocities Same mixture as 19, at 20 mm Hg.

**Figure 23:**
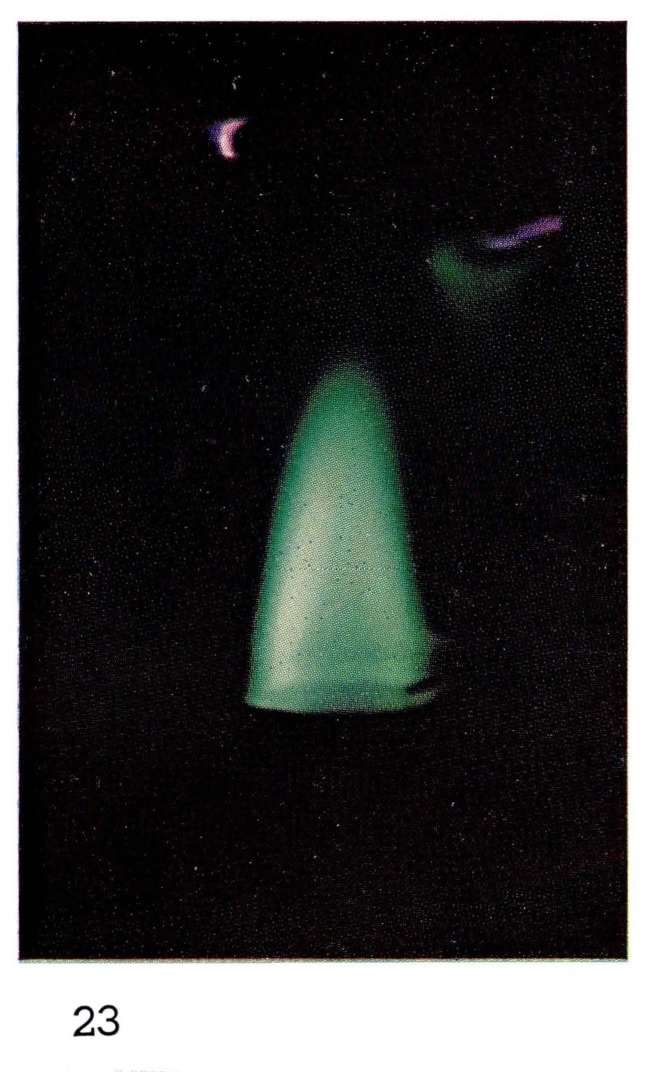
Flames Metastable helium with added oxygen.

**Figure 24:**
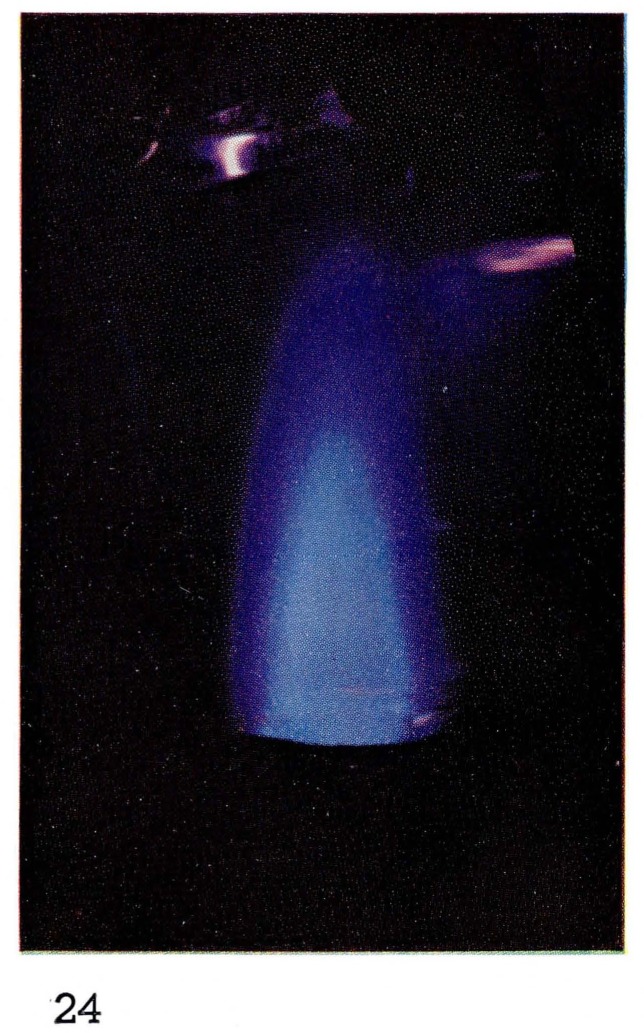
Flames Metastable helium with added nitrogen.

## Footnotes

1 Figures in brackets indicate the literature references at the end of this paper.
